# Sex and *APOE*: A memory advantage in male *APOE* ε4 carriers in midlife

**DOI:** 10.1016/j.cortex.2016.12.016

**Published:** 2017-03

**Authors:** Nahid Zokaei, Kathrin Giehl, Annie Sillence, Matt J. Neville, Fredrik Karpe, Anna C. Nobre, Masud Husain

**Affiliations:** a Oxford Centre for Human Brain Activity, Department of Psychiatry, University of Oxford, Oxford, UK; b Department of Experimental Psychology, University of Oxford, Oxford, UK; c Department of Nuclear Medicine, University of Cologne, Cologne, Germany; d Centre for Diabetes, Endocrinology and Metabolism (OCDEM), University of Oxford, Churchill Hospital, Headington, Oxford, UK; e NIHR Oxford Biomedical Research Centre, ORH Trust, Oxford, Churchill Hospital, Oxford, UK; f Nuffield Department of Clinical Neurosciences, University of Oxford, Oxford, UK

**Keywords:** Working memory, Alzheimer's disease, ApoE, Binding

## Abstract

Short-term memory in *middle-aged* individuals with different *APOE* alleles was examined using a recently developed task which is sensitive to medial temporal lobe (MTL) damage. Individuals (age-range: 40–51 years) with ε3/ε3, ε3/ε4 and ε4/ε4 *APOE* genotypes (*N* = 60) performed a delayed estimation task with a sensitive continuous measure of report. The paradigm allowed us to measure memory for items and their locations, as well as maintenance of identity-location feature binding in memory. There was a significant gene-dosage dependent effect of the ε4 allele on performance: memory decay or forgetting was slower in ε4 carriers, as measured by localization error and after controlling for misbinding errors. Furthermore ε4 carriers made less misbinding errors. These findings were specific to male carriers only. Thus, male ε4 carriers are at a behavioral advantage in midlife on a sensitive task of short-term memory. The results would be consistent with an antagonistic pleiotropy hypothesis and hightight the interaction of gender on the influence of *APOE* in cognition.

## 1 Introduction

Alzheimer's disease (AD) is a progressive neurodegenerative disorder associated with cognitive decline. Approximately 13% of individuals over the age of 65 years and 45% above the age of 85 are diagnosed with AD (AssociationA. ’s, 2016, Liu et al., 2013). The apolipoprotein E (*APOE*) ε4 allele confers the highest known genetic risk for developing AD, with increased frequency and lower age of onset occurring in a gene dose-dependent manner (Liu et al., 2013). Although the estimated prevalence of *APOE* ε4 allele in the normal population is approximately 12% (Myers et al., 1996), it has been estimated that 40–65% of those diagnosed with AD have one or two copies of the *APOE* ε4 allele (Saunders et al., 1993). But why would such a seemingly deleterious allele survive in the population?

One possibility is that carriers of the *APOE* ε4 allele might actually be at an advantage earlier in life, with detrimental traits associated with the allele evident only at a point beyond normal reproductive age – an example of what is termed antagonistic pleiotropy in evolutionary biology (Williams, 1957). In fact, it has been suggested that the possession of the APOE ε4 allele may result in cognitive advantage in younger carriers, but cognitive impairment in the old age (Tuminello & Han, 2011). In line with this hypothesis, a few studies have shown that older carriers of the *APOE* ε4 demonstrate poorer cognitive performance independent of AD pathology (Small et al., 2004, Wisdom et al., 2011) while young adults and children carriers of the *APOE* ε4 can demonstrate better cognitive performance (Jochemsen et al., 2012, Mondadori et al., 2007, Rusted et al., 2013).

It is unclear though whether the advantageous effects of *APOE* ε4 allele are present in *middle-age* (for a review see Salvato, 2015). It has been suggested from twin studies that the genetic effects on cognition increase with age (McClearn et al., 1997, McGue and Christensen, 2002) and hence should be more obvious during middle compared to young age. The evidence for the effect of *APOE* ε4 allele on cognition in midlife however, is mixed. While some studies have reported negative findings (Flory et al., 2000, Greenwood et al., 2005, Levy et al., 2004), others have found positive (Evans et al., 2014, Jochemsen et al., 2012) or null (Nilsson et al., 2006, Reiman et al., 1996, Sager et al., 2005) effects on cognitive functions such as attention, memory and executive function.

Various factors may contribute to the inconsistency in the literature. Most studies use standardized neuropsychological tests (McGue and Christensen, 2002, Nilsson et al., 2006, Reiman et al., 1996), often used as diagnostic tools to detect cognitive impairment in patient populations. In middle-aged healthy individuals, however, any effect of the *APOE* ε4 allele is likely to be subtle and hence probably not detected by such coarse measures. Another contributing factor to inconsistent finding comes from a possible interaction between gender and *APOE* genotype on cognition. Recent studies have reported significantly greater risks of developing AD and rates of cognitive decline in female carriers of the APOE ε4 (e.g., Altmann et al., 2014, Kozauer et al., 2008, Lin et al., 2015). Further, the possible presence of older carriers of the *APOE* ε4 allele with preclinical, undiagnosed AD in the broad age range of middle-aged participants (40–65 years) might confound separation of possible protective effects of *APOE* on cognition from prodromal AD.

Here, we sought to test the hypothesis that *APOE* status affects memory in middle-age, differently in males and females, using a sensitive task of short-term memory. Our middle-aged group had a mean age of 45, well outside the likely onset of prodromal AD, in most individuals (assuming more than 10 years for the prodromal stage and mean age of diagnosis being well above 60 in the general population, Wilson, Leurgans, Boyle, & Bennett, 2011). All participants completed a recently developed delayed estimation task of visual short-term memory, sensitive to medial temporal lobe (MTL) damage (Pertzov et al., 2013). Both patients with focal MTL lesions and familial AD due to genetic mutations in presenilin 1 or amyloid precursor protein genes show a specific pattern of short-term memory deficit: misbinding object identity with object location over brief intervals of time (Liang et al., 2016, Pertzov et al., 2013). In those previous studies, both MTL-lesion patients and pre-symptomatic familial AD cases showed poorer overall localization memory. Furthermore, all their deficits in remembering the locations of correctly identified objects were attributable to ‘swap errors’: they mislocalized a remembered item to the location of one of the other items they were holding in memory. Such misbinding of visual feature information has also been demonstrated in AD, both sporadic and familial, using other visual short-term memory paradigms (Parra et al., 2009, Parra et al., 2010, Parra et al., 2015).

Tasks similar to the one deployed here measure the resolution with which items are maintained in memory. This contrasts with classical neuropsychological measures of span, which quantify the number of items that can be retained. Delayed estimation tasks have been shown to be more sensitive to subtle differences in memory performance than span measures (Rolinski et al., 2014, Zokaei et al., 2015). We compared performance on our task across 3 different genotypes of the *APOE* gene: ε3/ε3, ε3/ε4 and ε4/ε4. In line with the antagonistic pleiotropy hypothesis we found a dose-dependent genetic enhancement for memory performance, but only in male carriers of the *APOE* ε4 allele.

## 2 Materials and method

### 2.1 Participants

A total of 60 individuals were selected from the Oxford Biobank, which consists of a sample of 3337 healthy volunteers (40–50 years) from Oxfordshire. For the present study, participants with *APOE* ε3/ε3, ε3/ε4 and ε4/ε4 genotype (20 individuals per group) were invited to participate by post. The first 20 positive replies, per genotype, were then invited to attend for memory testing. Considering that *APOE* alleles are not uniformly distributed in the population, number of volunteers with APOE ε3/ε3 was higher than other groups. To avoid the experimenter from guessing the genotype of each group, the person recruiting the participants was not involved in testing, and ensured equal number of participants per group were recruited/scheduled for testing at any given time. Neither the experimenter nor the participant was aware of the genetic status at the time of testing (double-blind protocol). All participants had normal or corrected-to-normal visual acuity and normal color vision (see Table 1 for demographics). This study was approved by University of Oxford Research Ethics Committee.

The groups of individuals with different APOE alleles did not differ in age [*F*(2,59) = .4, *P* > .6], gender [χ^2^(2,*N* = 60) = .9; *P* = .6] or handedness [χ^2^(2,*N* = 60) = 3.7; *P* = .15].

### 2.2 *APOE* genotyping

*APOE* genotyping was carried out using Applied Bio-system, Assay-on-demand TaqMan^®^ SNP genotyping Assays, C_3084793_20 and C_904973_10 corresponding to APOE SNPs rs429358 and rs7412, respectively, and run on an ABI 7900HT Fast Real-Time PCR system. Haplotypes corresponding to APOE ε3 and ε4 were then deduced.

### 2.3 Cognitive assessment

The Addenbrooke's Cognitive Examination (ACE III) test was administered as a general cognitive screening test to all participants. The ACE is a brief neuropsychological assessment of cognitive function with five main domains of attention, memory, fluency, language and visuo-spatial abilities widely used for identifying mild cognitive impairment and dementia. Mean scores for each group are given in Table 1. None of the participants exhibited significant cognitive impairment as measured by the ACE, using a cut-off score of 88/100. Further, there was no difference in ACE scores between the groups of individuals with different *APOE* alleles [*F*(2,59) = 1.03, *P* > .3].

### 2.4 Visual short-term memory task

#### 2.4.1 Procedure

**Fig. 1:**
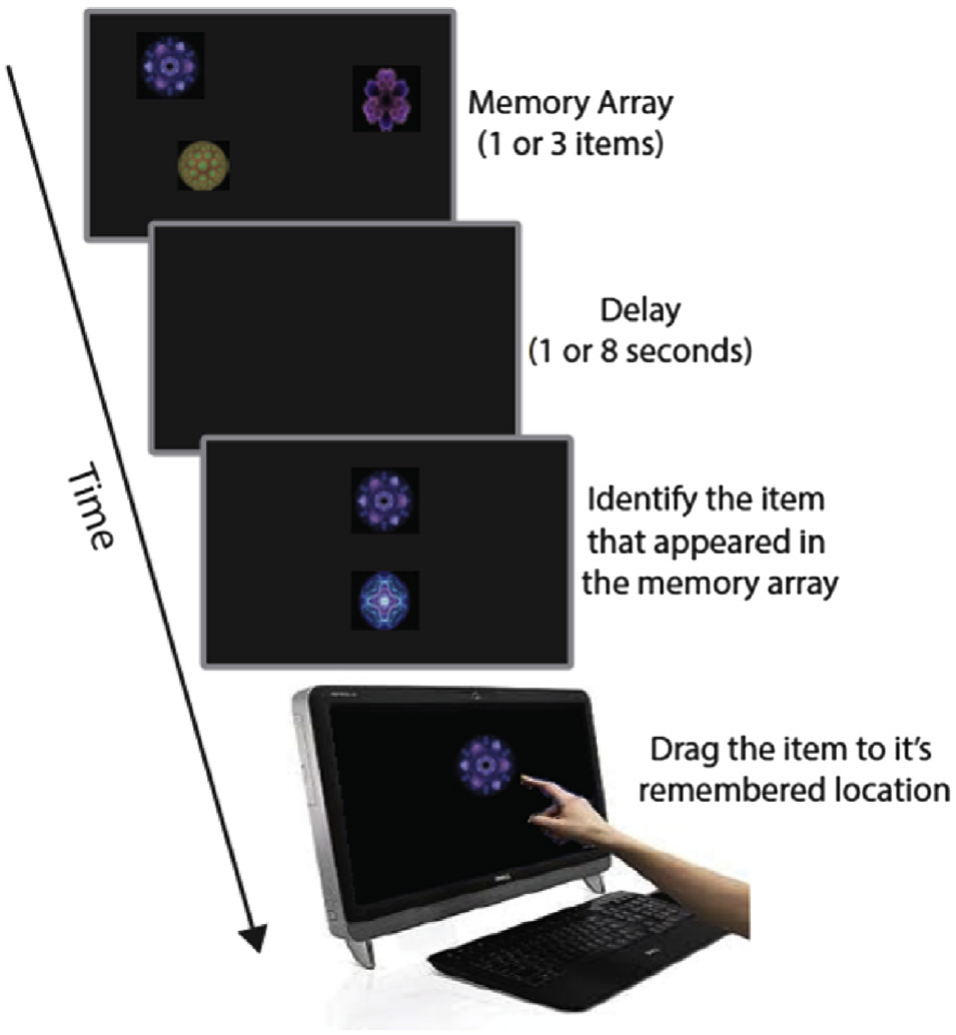
Visual short-term memory task.

Participants performed a visual short-term memory task, identical to that previously used by Pertzov et al. (Pertzov et al., 2013). A schematic representation of the task is illustrated in Fig. 1. Briefly, in each trial participants were presented with 1 or 3 fractals objects (the memory array) for 1 or 3 sec respectively (to ensure thorough encoding) and asked to remember both the identity and the location of the these objects. This was followed by a delay of either 1 or 8 sec before recall phase of the study. The recall phase consisted of two steps. Participants were first presented with 2 fractals on the vertical meridian of the screen. One of these had been present in the preceding memory array while the other was a foil or distractor (i.e., an object that did not appear in the original memory display). Participants were required to select the fractal they remembered by touching it – *identification step* – and then drag it to its remembered location on a touchscreen computer – *localization step*. They confirmed their response with a key press before the start of the next trial. The task therefore provides memory measures for both object identity and location separately, but note that while the identification is a binary report measure (correct/incorrect response) the localization provides a continuous, analog measure of response accuracy or precision.

Participants were presented with 1 or 3 fractals followed by a 1 or 8 sec delay. They were then presented with two fractals, one from the memory set (target) and a foil. On a touchscreen computer, they first had to touch the fractal they had seen before in the memory array and then drag it to its remembered location. This allowed measurement of memory for object identification and localization error. Sometimes participants recalled the object correctly but moved it to the location of one of the other items they had seen in the memory array, making a ‘swap’ or misbinding error.

Participants performed 2 blocks of 50 trials each. Each block included 16 trials in which one fractal was presented. Half the trials had a 1-sec delay between memory array presentation and probe, while the other half had an 8-sec delay. In addition, there were 34 trials in which 3 fractals were presented in the memory array (half with 1 sec and half with 8 sec delay). Prior to the start of the experiment, participants were acquainted with the experimental apparatus and conditions by gradually increasing the complexity of the practice trials. All participants completed 10 practice trials in total.

#### 2.4.2 Stimuli and apparatus

Stimuli were presented on a touchscreen (Inspiron All-in-One 2320; DELL) with a 1920 × 1080 pixel resolution (corresponding to 62° × 35° of visual angle) at a viewing distance of approximately 42 cm. The stimuli were randomly selected from a pool of 60 fractals (http://sprott.physics.wisc.edu/fractals.htm). Each fractal was presented 2 to 3 times per block. The fractals had a maximal width and height of 120 pixels (4° of visual angle). The location of each fractal was determined in a random manner but with the following restrictions: all fractals had a minimum distance of 9° of visual angle from each other (to avoid spatial crowding) and each fractal had a minimum distance of 3.9° of visual angle from the edges of the screen and a minimum distance of 6.5° from the center of the screen.

#### 2.4.3 Analysis

Identification and localization accuracies will be assessed using repeated measures ANOVAs with number of items in memory array (1 or 3 fractals) and delay (1 or 8 sec) as within subject factors and APOE status (ε3/ε3, ε3/ε4 and ε4/ε4) and gender (male and female) as between subject factors (please refer to Supplementary Material for results with carrier vs. non-carriers of APOE ε4 as between subject factor). Due to small number of participants per gender group, we first report the findings in ANOVAs performed without gender as the between subject factor and later add this factor to the analysis. Any follow-up ANOVAs will only be performed if any interactions with within subject factors reaches significance.

## 3 Results

### 3.1 Identification performance

We first analyzed identification performance, i.e., the frequency with which participants picked the correct fractal in the two-alternative forced-choice identification step. This is a binary report (correct/incorrect) measure. Repeated measures ANOVA, with number of items (1 or 3 items) and delay (1 or 8 sec) as within-subject factors and *APOE* allele as a between-subjects factor was used. There was a significant effect of set size [*F*(1,57) = 115, *P* < .001, *η*^2^_*P*_ = .67] as well as of delay [*F*(1,57) = 12, *P* = .001, *η*^2^_*P*_ = .17]. In addition, there was a significant interaction between delay and set size [*F*(1,57) = 15, *P* < .001, *η*^2^_*P*_ = .22], with reduced identification performance for longer delays occurring with 3 fractals vs. 1 fractal. There was no significant effect of *APOE* status [*F*(2,57) = .46, *P* = .6, *η*^2^_*P*_ = .01] on identification performance.

### 3.2 Localization performance

**Fig. 2:**
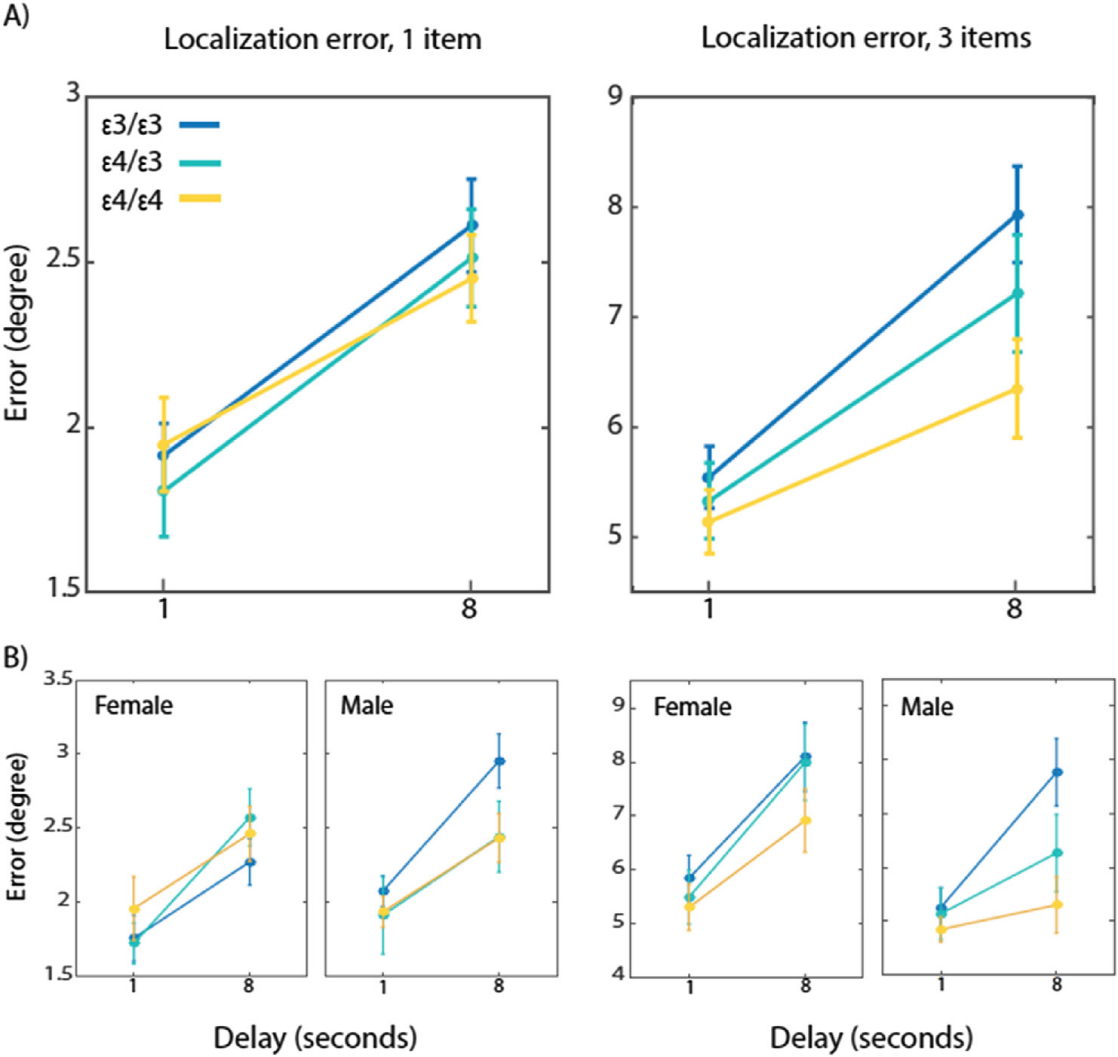
Localization performance according to genotype and gender.

Next we examined *localization* memory by measuring the distance between true and reported locations. Thus this provides a continuous report or analog index of memory. For this step of the analysis, only trials where participants had previously picked the correct item in the identification phase were included. Localization performance was worse with larger set sizes and longer delays [main effects *F*(1,57) = 443, *P* < .001, *η*^2^_*P*_ = .89 and *F*(1,57) = 112, *P* < .001, *η*^2^_*P*_ = .66 respectively; Fig. 2A]. Furthermore, the interaction between delay and set size was consistent with significantly increased error for set size 3 and delay of 8 sec [*F*(1,57) = 29, *P* < .001, *η*^2^_*P*_ = .34]. In this analysis, *APOE* status interacted marginally with delay [*F*(2,57) = 3.9, *P* = .059, *η*^2^_*P*_ = .1]. Follow-up analysis demonstrated no further significant main effects or interaction.

### 3.3 Gender affects localization performance

We next added gender of participants as a between subject factor. Gender interacted significantly with set size [*F*(1,54) = 7, *P* = .01, *η*^2^_*P*_ = .12] while *APOE* status interacted significantly with delay [*F*(2,54) = 3.8, *P* = .028, *η*^2^_*P*_ = .13]. Follow-up analysis demonstrated that *APOE* status interacted with delay only in males [*F*(2,23) = 6.33, *P* = .006, *η*^2^_*P*_ = .35] and not in female participants [*F*(2,31) = 1.26, *P* = .298, Fig. 2B, refer to Fig. S1 for individual participant performance per APOE status and gender].

Memory resolution for location was calculated as the difference in angular degrees between the target location and the response for different memory set sizes (1 or 3 items) and delays (1 or 8 sec). Results are shown for all individuals (**A**) and broken down by gender (**B**). **A**) Memory decay was slower in carriers of the APOE ε4 gene, in a gene-dosage manner. **B**) Memory decay for location was slower in male *APOE* ε4/ε4 carriers than male ε4/ε3 or ε4/ε4 carriers, particularly when 3 items had to be retained in memory for 8 sec. There was no significant effect of delay on memory performance in female participants.

Findings from the analysis of localization error raise a critical question: Is less decay of memory in the *APOE* ε4 carriers simply due to preserved memory for location, better binding of identity to location, or both?

### 3.4 Reduced swap errors in memory in ε4 carriers

We first examined the effect of *APOE* status ε4 on maintenance of bound objects (identity of fractal to locations) in short-term memory. To test this we first counted the frequency with which participants localize the fractal around 4.5° of one of the *non-probed* fractal locations, i.e., one of the other object locations shown in the memory array (see Pertzov et al., 2013 for detailed description of method). There was a significant interaction between delay and *APOE* status in proportion of such swap errors [*F*(1,54) = 4.7, *P* = .013, *η*^2^_*P*_ = .15] and a significant main effect of gender on proportion of swap errors [*F*(1,54) = 6.69, *P* = .012, *η*^2^_*P*_ = .11]. *APOE* status interacted significantly with delay in male [*F*(2,23) = 6.95, *P* = .004, *η*^2^_*P*_ = .377] and not female participants (*P* > .1). There was no significant difference in proportion of swap errors in male participants between different *APOE* genotypes following a 1 sec delay (*P* > .05) but reached uncorrected significance following an 8 sec delay between APOE ε4/ε4 and ε3/ε3 carriers [*t*(21) = 2.23, *P* = .037].

### 3.5 Decay in memory precision

Can reduced swap errors with longer delays in *APOE* ε4 carriers explain their overall superior localization performance (Fig. 2)? In other words, is the reduced overall error observed in memory for location attributable to making fewer swap errors? To answer this question, we calculated localization error not by measuring the distance to the correct location of the probed item but as the distance between the reported location and the nearest original location of *any* of the 3 items shown in the memory display. This type of measurement has been called a ‘nearest neighbour’ control analysis (Pertzov et al., 2013). Effectively, this analysis controls for swap errors since in trials where a swap occurs, the location of the non-probed item is now treated as if it was the probed location.

**Fig. 3:**
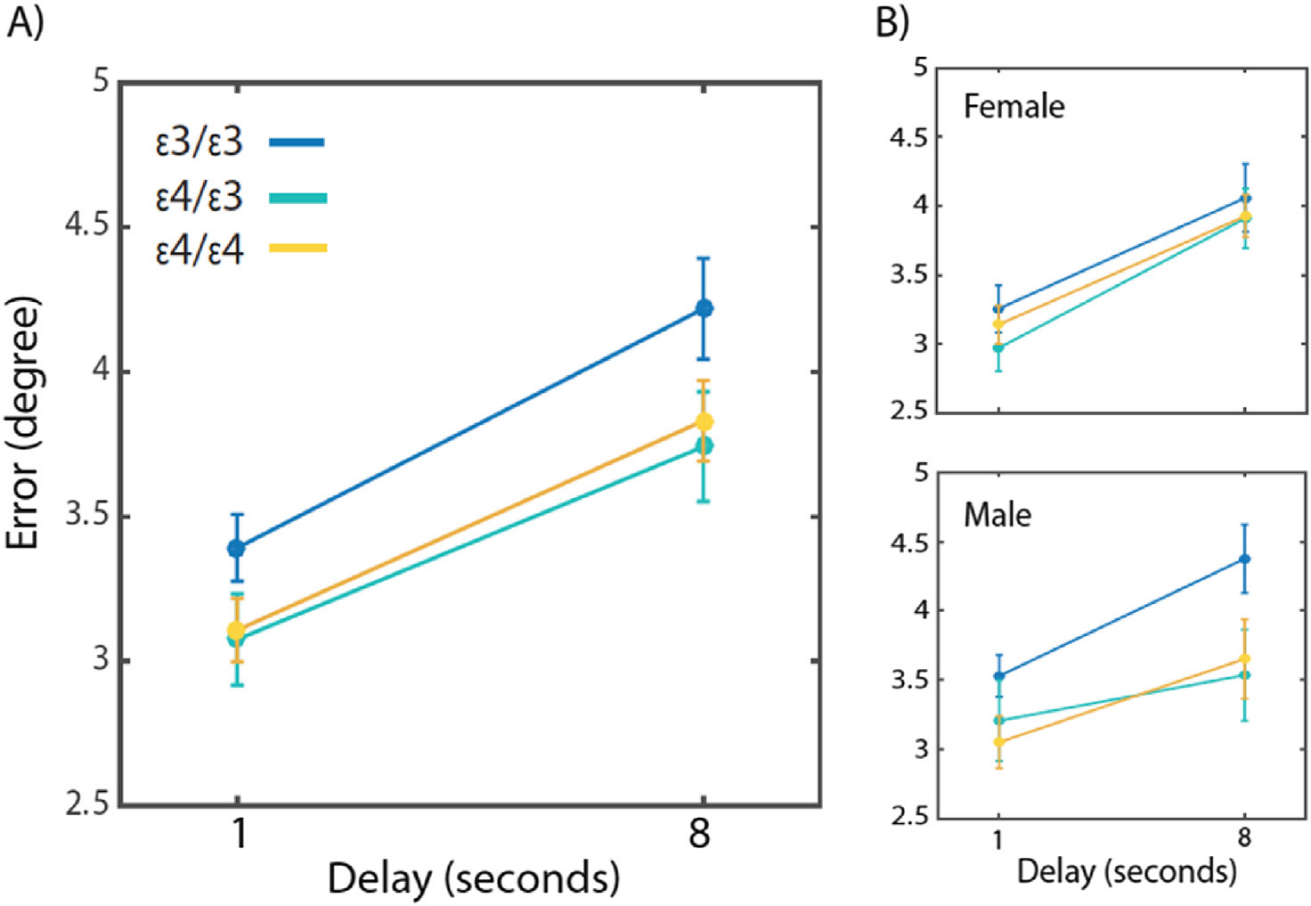
Nearest neighbour control analysis for the 3-item condition.

Localization error after controlling for swap errors – here referred to as nearest neighbor control – in an ANOVA revealed a significant main effect of set size [*F*(1,54) = 437, *p* < .001, *η*^2^_*P*_ = .89], delay [*F*(1,54) = 230 *p* < .001, *η*^2^_*P*_ = .8, Fig. 3A] and a significant interaction between delay, *APOE* status and gender [*F*(2,54) = 4.87, *p* = .011, *η*^2^_*P*_ = .15]. Follow-up analysis revealed a significant interaction between delay and *APOE* status, but in male participants only [*F*(2,23) = 4.6, *p* = .02, *η*^2^_*P*_ = .29, Fig. 3B].

This analysis controls for swap errors to examine if any differences between groups is due only to swap errors. Data shown for all participants (A) and by gender (B). After controlling for swap errors, there was no difference in the performance of females in any of the groups. However, male carriers of the APOE ε4 (both ε4/ε4 and ε4/ε3 groups) performed better compared to non-carriers.

In summary, the behavioral advantage of carriers of male *APOE* ε4 allele can be explained firstly by a decrease in proportion of responses to one of the non-probed items held in memory-errors attributed to misremembering the correct location of items in memory. Maintenance of binding of features in short-term memory has been linked to hippocampal function (Liang et al., 2016, Pertzov et al., 2013) and in our study male carriers of the *APOE* ε4 allele made fewer of these errors compared to healthy controls. Moreover *APOE* ε4 carriers also demonstrated a slower decay of feature memory – in our study that is memory for spatial locations – after controlling for swap errors. Together, they give rise to enhanced memory performance in the male *APOE* ε4 carrier groups. Similar pattern of findings were observed when looking at APOE ε4 carriers vs. non-carriers, i.e., 2 groups of APOE status instead (see supplementary material).

## 4 Discussion

In the current study we examined in middle-aged participants the influence of the *APOE* gene on performance in a visual short-term memory task. Overall, we found evidence for significantly enhanced short-term memory performance in carriers of the *APOE* ε4 allele. More specifically our findings demonstrate a gene-dosage dependent influence of the ε4 allele on performance; short-term representations of object location decayed more slowly in carriers of the ε4/ε4 followed by those with ε3/ε4 and ε3/ε3 (Fig. 2) alleles. Importantly, such a pattern of performance was only present in males. These findings, to the best of our knowledge, are the first to demonstrate a cognitive *advantage* of the *APOE* ε4 allele exclusively in middle-aged males. The results would be consistent with an antagonistic pleiotropy account of why the *APOE* ε4 allele has survived despite its evident deleterious effect in later life (Tuminello and Han, 2011, Williams, 1957).

Only a few previous studies have investigated the effect of APOE on short-term memory in middle-aged individuals. In one, Greenwood and colleagues tested participants' ability to retain spatial location for brief periods of time, in a group of individuals between the ages 41 and 85 years of age. *APOE* ε4/ε4 exerted deleterious effects on spatial short-term memory, only in conditions with the highest memory load, i.e., larger set size (Greenwood et al., 2005). Paradoxically, however, a longitudinal study with an identical design reported increased accuracy in middle-aged (mean age of 50) and impaired performance in older carriers of *APOE* ε4 (mean age of 65) across three years in midlife (Greenwood, Espeseth, Lin, Reinvang, & Parasuraman, 2014). In both studies, the age-range of participants was on average around 5 years higher than the present study, the effect of gender was not examined and, importantly, the task used may not have been sensitive to subtle differences between groups and across time. The paradigm required participants to make only a binary response: either a location was remembered correctly or it was not. It is important to note that just because an individual fails to recall an item, it does not necessarily mean that information regarding that item was completely lost from memory (Ma, Husain, & Bays, 2014). The design of the current study overcomes this issue by measuring both the identity of the objects, the resolution with which locations were maintained in a continuous analog manner, and the binding between these two sorts of information. This type of task has been shown to be more sensitive than traditional discrete measures of short-term memory such as digit span (Zokaei et al., 2015).

Using a continuous estimation method we found that memory decay for object location in this middle-aged group was less in ε3/ε4 and ε4/ε4 groups compared to the ε3/ε3 group. This effect was strongest in the more demanding trials, i.e., those with 3 items to be retained in memory (although a similar but weaker effect was observed for set size 1). Interestingly, this reduced decay was explained by both a decrease in number of swap errors (errors attributed to misbinding of item identity to correct location) as well as better memory resolution for location (regardless of swap errors) in ε4 carriers. Maintenance of bound information has been shown to rely on the MTL (Liang et al., 2016, Pertzov et al., 2013). Although behavioral reports in line with our findings are scarce, imaging studies have shown subtle differences between middle-aged ε4 carrier and non-carriers. Middle-aged ε4 carriers have enhanced functional connectivity between the default mode network and frontal, parietal and temporal cortical regions, with increased connectivity associated with better memory (Goveas et al., 2013). It is possible that some form of compensatory mechanism such as this might be present in middle-aged ε4 carriers resulting in better memory resolution and binding. Recently, in line with such a proposal, Dowell and colleagues reported greater connectivity and white matter volume in young *APOE* ε4 carriers compared to non-carrier and middle-aged participants alongside a positive correlation with behavioral memory measures. Such findings may suggest the possibility of an over engagement of some brain regions in ε4 carriers with negative consequences later in life (Dowell et al., 2016). It's important to note however, that middle-aged participants in their study were on average 5 years older than those who participated in this study.

The most intriguing finding from the present study was that beneficial effects of ε4 status were observed mostly only in male and not female participants. The influence of gender on *APOE* effects is an important observation in the context of several other reports. Female carriers of the *APOE* ε4 are more likely to develop AD (Farrer et al., 1997) and show significantly greater hippocampal atrophy (Fleisher et al., 2005). One possible mechanism for the effect of gender in AD is the role of sex hormones on brain and cognitive function, apparent in both animal and human studies investigating the role estrogen on risk of dementia and cognition in women (for review, Farrer et al., 1997) as well as the importance of *APOE* on evaluating estrogen replacement (Kang and Grodstein, 2012, Yaffe et al., 2000). It is possible that sex hormones could influence the interaction between genetic factors and cognitive performance, resulting in enhanced performance in male carriers.

Regardless of the mechanism, the findings reported here suggest that male carriers of the *APOE* ε4 allele might actually be at an advantage earlier in life, with detrimental traits associated with the allele becoming evident only at a point beyond reproductive age – an example of what is termed antagonistic pleiotropy in evolutionary biology (Williams, 1957). Thus although the risk of AD is increased ∼15 times for ε4/ε4 individuals, who virtually all have AD by the age of 80 (Liu et al., 2013), this would not have been an important factor for evolutionary fitness because human life expectancy was far less until relatively recently. Most individuals would have died before AD developed. Indeed, if male ε4 carriers are at an advantage when they are reproductively fit there might even have been a selection advantage for them (Tuminello & Han, 2011).

In this study, by testing middle-aged individuals, we minimized any influences of early processes associated with undetected AD, a criticism of most studies with older participants. Because of the broad age range of middle-aged participants in the few studies that have been conducted previously, a distinction between prodromal AD and beneficial effect of *APOE* genotype cannot definitively be made. This was minimized in our protocol by recruiting middle-aged individuals at least a decade prior to the mean prodromal stage of AD (Wilson et al., 2011). In addition, we used a sensitive cognitive task which uses a continuous, analog report which might have allowed us to demonstrate differences in performance that would not be visible to standard binary measures (correct/incorrect responses). Nevertheless, the findings reported here would need to be extended to a larger age range, and ideally longitudinally, to examine when the potential advantage of male ε4 carriers first becomes evident and whether it is lost at an older age.

## Figures and Tables

**Table 1:** Demographic characteristics of the final sample.

	Age Mean (SD)	Gender (m/f)	Handedness (R/L)	Years of Education Mean (SD)	ACE Mean (SD)
ε3/ε3	46.1 (2.7)	10/10	18/2	15.9 (3.8)	95.4 (2.9)
ε4/ε3	45.8 (2.7)	9/11	15/5	15.3 (2.7)	95.7 (3.8)
ε4/ε4	45.3 (3.3)	7/13	19/1	15.6 (2.8)	96.8 (2.6)
