# Supplementary material for: Sex and APOE: A memory advantage in male APOE ε4 carriers in midlife
Source: Cortex. 2017 Mar;88:98–105. doi: 10.1016/j.cortex.2016.12.016 (PMC5333781; doi:10.1016/j.cortex.2016.12.016)
Supplement: Supplementary file 1 [file mmc1.docx]

**Results**

Memory performance between carrier (n=20) and non-carrier (n=40) of the ε4 allele.

**Identification performance**

Repeated measures ANOVA, with number of items (1 or 3 items) and delay (1 or 8 seconds) as within-subject factors and *APOE* allele (either carrier or non-carrier of the APOE ε4) as a between-subjects factor was used. There was a significant effect of memory set size (*F*(1,58)=98.5, *p*<0.001, η^2^_p_=0.63) as well as of delay (*F*(1,58)=12.6, *p*=0.001, η^2^_p_=0.179). In addition, there was a significant interaction between delay and set size (*F*(1,58)=12.9, *p*=0.001, η^2^_p_=0.18), with reduced identification performance for longer delays occurring with 3 fractals vs. 1 fractal. There was no significant effect of *APOE* status (*F*(1,58)=0.14, *p*=0.7, η^2^_p_=0.02) on identification performance.

**Localization performance**

Next, we examined the effect of APOE status on localization error. There was a significant main effect of memory set size (*F*(1,58)=411, *p*<0.001, η^2^_p_=0.87), delay (*F*(1,58)=110, *p*<0.001, η^2^_p_=0.65) and a significant interaction between the two factors(*F*(1,58)=32.5, *p*<0.001, η^2^_p_=0.36).

However, when adding gender as a between subject factor, there was a significant interaction between delay and APOE status (*F*(1,56)=4.8, *p*=0.033, η^2^_p_=0.08). Furthermore, gender interacted with set size (*F*(1,56)=5.9, *p*=0.018, η^2^_p_=0.096) and there was a significant 3-way interaction between delay, APOE status and gender (*F*(1,56)=4, *p*=0.048, η^2^_p_=0.07). Following-up on the interaction, APOE status interacted with delay only in male (*F*(1,24)=11.8, *p*=0.002, η^2^_p_=0.33) and not female participants (*p*>0.05). Furthermore, there was a significant 3-way interaction between APOE status, gender and delay (*F*(1,24)=4.25, *p*=0.050, η^2^_p_=0.15) and a main effect of APOE status (*F*(1,24)=4.5, *p*=0.044, η^2^_p_=0.16) in male participants.

**Proportion of Swap errors**

There was a significant main effect of delay (*F*(1,56)=5.55, *p*=0.022, η^2^_p_=0.09) and gender (*F*(1,56)=7.99, *p*=0.007, η^2^_p_=0.12) and a significant interaction between delay and APOE status (*F*(1,56)=5.9, *p*=0.018, η^2^_p_=0.096) on proportion of swap errors. Follow-up analysis demonstrated no further significant main effects or interaction.

**Nearest Neighbor control**

Localization error after controlling for swap errors demonstrated a significant main effect of set size (*F*(1,56)=443, *p*<0.001, η^2^_p_=0.88) and delay (*F*(1,56)=213, *p*<0.001, η^2^_p_=0.79) and a significant interaction between APOE status and set size (*F*(1,56)=4.9, *p*=0.030, η^2^_p_=0.082). Moreover, there was a significant 3-way interaction between gender, APOE status and delay (*F*(1,56)=3.99, *p*=0.050, η^2^_p_=0.067). Follow-up ANOVAs demonstrated a significant interaction between delay and APOE status and a significant main effect of APOE status in male (*F*(1,24)=8.9, *p*=0.006, η^2^_p_=0.27 and *F*(1,24)=4.3, *p*=0.048, η^2^_p_=0.14) and not female participants (*p*>0.05). Moreover, APOE status interacted with set size significantly in female (*F*(1,32)=4.9, *p*=0.034, η^2^_p_=0.13) and not male participants (*p*>0.05). Follow-up t-tests revealed uncorrected significant differences in error between carrier and non-carriers of the APOE ε4 in trials following longer delays, in both trials with 1 and 3 fractals (*t*(24)= 2.169, *p*=0.04 and *t*(24)= 2.35, *p*=0.028 respectively).


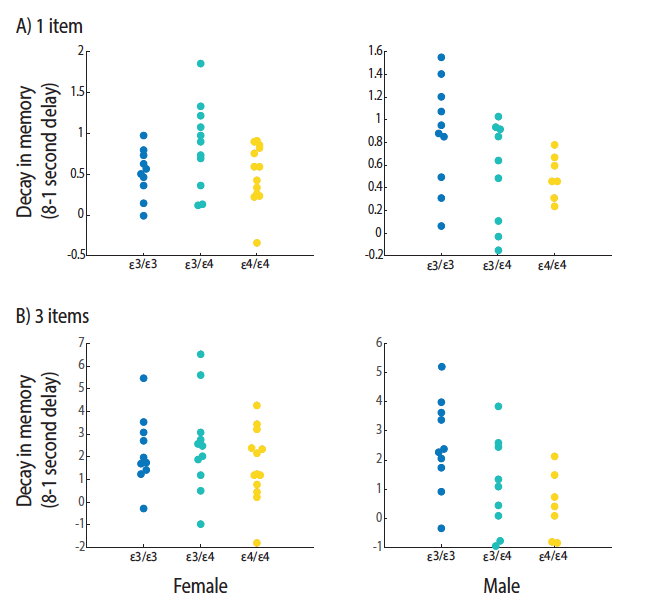


**Figure S1**- Change in localization performance from 1 and 8 seconds delay in each set size condition, per gender and APOE status group. Smaller values correspond to slower decay of memory.
